# Production and Expression Optimization of Heterologous Serratiopeptidase

**Published:** 2020-05

**Authors:** Maryam ROUHANI, Vahideh VALIZADEH, Sara MOLASALEHI, Dariush NOROUZIAN

**Affiliations:** Department of Nano-Biotechnology, New Technologies Research Group, Pasteur Institute of Iran, Tehran, Iran

**Keywords:** Serratiopeptidase, Cloning, Expression optimization

## Abstract

**Background:**

Serratiopeptidase is a bacterial metalloprotease, which is useful for the treatment of pain and inflammation. It breaks down fibrin, thins the fluids formed during inflammation and acts as an anti-biofilm agent. Because of medicinally important role of the enzyme, we aimed to study the cloning and the expression optimization of serratiopeptidase.

**Methods:**

The heat-stable serratiopeptidase (5d7w) was selected as the template. Cloning into pET28a expression vector was performed and confirmed by colony PCR and double restriction enzyme digestion. The recombinant protein was expressed in *Esherichia coli* BL21 and confirmed by SDS-PAGE and Western blot analysis. Different parameters such as expression vector, culture media, post-induction incubation temperature, inducer concentration, and post-induction incubation time were altered to obtain the highest amount of the recombinant protein.

**Results:**

Serratiopeptidase was successfully cloned and expressed under optimized conditions in *E. coli* which confirmed by western blot analysis. The optimal conditions of expression were determined using pQE30 as vector, cultivating the host bacteria in Terrific Broth (TB) medium, at 37° C, induction by IPTG concentration equal to 0.5 mM, and cells were harvested 4 h after induction.

**Conclusion:**

As serratiopeptidase is a multi-potent enzyme, the expressed recombinant protein can be considered as a valuable agent for pharmaceutical applications in further studies.

## Introduction

Serratiopeptidase is one of the most important proteolytic enzymes derived from nonpathogenic *Serratia marcescens* sp. E15. Historically, this proteolytic enzyme was isolated from silkworm *Bombyx mori* intestine in the 1960s (1,2). Serratiopeptidase is also known as serrapeptase or serralysin, has been used for treatment of sinusitis, carpal tunnel syndrome, bronchitis, atherosclerosis, tissue permeation enhancement activity, wound debridement, post-traumatic swelling and fibrocystic breast disease. Clinical studies have shown that it can digest dead tissues, blood clots, cysts, and arterial plaques (3–6). Owing to lack of side effects, the enzyme is of rational choice as a substitute for harmful non-steroidal anti-inflammatory agents (NSAIDs). It reduces exudates viscosity and inhibits hydrolysis of inflammatory mediators such as histamine, bradykinin and serotonin. Moreover, recent studies showed serratiopeptidase can reduce the biofilms produced by some bacteria (7,8).

Serratiopeptidase is a single-chain protein possessing 470 amino acids (Serra 470) with a molecular weight of about 50 kDa (9). The enzyme belongs to metzincins superfamily, which also includes other proteases such as alkaline proteases from *Pseudomonas aeruginosa* and protease C from *Erwinia chrysanthemi* (10,11). The X-ray crystal structures of the enzyme revealed that calcium ions bind to the conserved aspartate/glycine-rich repeats, which lead to protein folding. Connection to the calcium carried out through interaction with carboxyl group of aspartate and carbonyl group of glycine. The catalytically active zinc is bound to the three histidines of the HEXXHXXGXXH motif (12).

Recombinant proteins provided important breakthroughs in medical biotechnology. They are not only used in biomedical research but also in treatment and prevention of diseases, as drugs and vaccine, respectively. Recombinant proteins could be produced easily as heterologous clones in different expression systems like bacteria, yeast, etc. (13). Consequently, the target protein should be cloned and produced in a way to obtain the correct structure with high yield and efficiency. Therefore, in the present study, Serra 470 was cloned and expressed in *E. coli*. Moreover, different expression protocols were screened out to select the best procedure to produce recombinant serratiopeptidase with high quantity and efficiency.

## Materials and Methods

### Cloning and expression of Serra 470

The last crystal structure of serratiopeptedase enzyme derived from bacterial strain *Serratia sp.* FS14 has been deposited in protein data bank (PDB) named as 5d7w in 2016 in China. The enzyme is a 50 kDa extracellular protein that retained 40% of its protease activity after incubation at 363 K for 10 min (14). Being resistance to environmental stresses like extreme temperatures is one of the high priorities in the enzyme development, thus 5d7w was selected for producing as recombinant protein. The gene encoding serratiopeptedase (5d7w) was extracted from NCBI and synthesized by Biomatic company (USA, Canada) after codon optimization. Then Serra 470 was cloned in pET28a vector (Qiagen, Germany) and expressed in *E. coli* BL21(DE3)pLysS*,* as an expression host. The recombinant clones were selected on LB (Luria-Bertani) agar plates containing kanamycin (25 μg/ml). Positive clones were confirmed by double digestion with restriction enzymes (*Nco*I and *Xho*I) and then analyzed by electrophoresis on 1% agarose gel. Finally, cloned fragments were then sequenced.

Serra 470 was expressed in *E. coli* BL21. Briefly, a single colony of the bacteria carrying Serra 470-pET28a recombinant plasmid was inoculated into 5 ml of LB broth containing kanamycin (25 μg/ml) incubated at 37 °C, 180 rpm for overnight. Overnight culture of Serra 470-pET28a was cultured in LB broth containing kanamycin (25 μg/ml) with shaking (180 rpm) at 37 °C until OD_600_ was reached to 0.6–0.8. The recombinant protein expression was induced with 0.5 mM isopropyl-β-D-thiogalactopyranoside (IPTG, Sigma, USA). The culture was grown further for 4 h and the cells were harvested by centrifuging at 7,000 rpm for 15 min., supernatant was discarded and pellet was kept at −20 °C until use.

In all steps, 1 ml of cultures were collected as pre-induction and post-induction for calculating growth by measuring optical density (OD) at 600 nm and analyzing the expressed protein by sodium dodecyl sulfate-polyacrylamide gel electrophoresis (SDS-PAGE, 12%).

### Optimization of Serra 470-pQE30

In this investigation, to increase amount of recombinant protein some independent expression parameters such as vector, culture media, post-induction incubation temperature, inducer concentration and post-induction incubation time were assessed and optimized. In each step, densitometry was performed to quantify the recombinant protein expression level using GS-800 calibrated densitometer (Biorad, USA) from SDS-PAGE-gels.

### Vector replacement

The first step for optimization for achieving high level of expression was vector replacement. In this regard, pQE30 vector was selected based on the previous successful experiences. To clone Serra 470 in pQE30 vector a set of primers were designed and Serra 470 was amplified with those designed primers that harbor binding sites for BamHI and HindIII restriction enzymes. The sequences of the primers were as follow:

**Forward Primer (Serra-pQE470F)**
- Annotation: Anchor BamHI gene sequence
- Sequence: ACGGGATCCGCGGCGGCGACC

**Reverse Primer (Serra-pQE470R)**
Annotation: Anchor HindIII gene sequence
Sequence: ACCCAAGCTTAACGATAAAGTCGG TC

Serra 470-pQE30 cloned and expressed as described above. Briefly, Overnight culture of *E. coli* M15 carrying Serra 470-pQE30 plasmid was cultured in LB media (pH 7.2) containing ampicillin (100 μg/ml) and kanamycin (25 μg/ml) at 37 °C with shaking (180 rpm) until an OD_600_ of 0.6-0.8 was reached. Then, the expression was induced by adding 0.5 mM IPTG. The culture was grown or further 4 h and the cells were harvested by centrifuging at 7,000 rpm for 15 min. The protein pattern of untransformed *E. coli* M15 (as a negative control) and Serra 470-pQE30- *E. coli* M15 was analyzed on SDS-PAGE, 12%.

### Optimization of culture media

To optimize the culture media, overnight culture of Serra 470-pQE30-*E. coli* M15 was transferred into four different media including: 2X LB, Terrific broth (TB, pH 7.2 containing: yeast extract 2.4%, glycerol 0.5%, tryptone 1.2%, K_2_HPO_4_, 0.072 M, KH_2_PO_4_, 0.017 M), 2X YT (tryptone 1.6%, yeast extract 1%, NaCl 0.5%) and Super optimal growth medium (SOB, containing: tryptone 2%, yeast extract 0.5%, NaCl 0.05%, KCl 0.01%, MgCl_2_ 0.09%, MgSO_4_ 0.12 %). Two antibiotics namely ampicillin (100 μg/ml) and kanamycin (25 μg/ml) were added to each medium before experiment. Bacterial cultures incubated at 37 °C with shaking (180 rpm) until the mid-exponential growth phase was achieved. Four hours after induction, the cells pellets kept at −20 °C until for analysis of expressed protein by SDS-PAGE.

### Optimization of post-induction incubation temperature

Overnight cultures of bacterial colonies containing Serra 470- pQE30 recombinant plasmid was transferred to fresh TB media (based on the results of previous section) containing ampicillin (100 μg/ml) and kanamycin (25 μg/ml) with shaking at 180 rpm and 37 °C. After induction with 0.5 mM IPTG at the mid exponential phase, bacterial cultures were incubated at three different temperatures; 25 °C, 30 °C and 37 °C at 180 rpm for 4 h. The cell pellets kept in −20 °C until analysis of expressed protein by SDS-PAGE.

### Optimization of inducer concentration

In this step, induction of *E. coli* M15 harboring Serra 470-pQE30 recombinant plasmid growing in TB medium were carried out at various concentration of IPTG (0.2 mM, 0.5 mM and 1 mM). Protein expression was done for 4h at 37 °C (based on the results of previous step), at 180 rpm. Cell pellets harvested until analysis of expressed protein by SDS-PAGE.

### Optimization of post-induction incubation time

To optimize the cell harvest time, as described previously, overnight culture of Serra 470-pQE30-E. coli M15 was expanded in TB media including ampicillin (100 μg/ml) and kanamycin (25 μg/ml) at 37 °C with shaking 180 rpm until reached OD of 0.6–0.8 at 600 nm. Then expression was induced by 0.5 mM IPTG (based on the results of previous section). The cell culture was further grown for 2 h, 4 h, 6 h, and overnight, then the cell pellets kept at −20 °C until analysis of expressed protein by SDS-PAGE.

### Confirmation of recombinant protein

To confirm the expressed recombinant protein, Western blot assay was carried out by standard protocols using anti-His antibody (Penta His Antibody; Qiagen). Because protein expressed using two mentioned vectors, pET28a and pQE30, carry a tail of poly histidines in the C or N terminal, respectively.

## Results

### Cloning and expression of recombinant Serra 470-pET28a-E. coli BL21

**Fig. 1:**
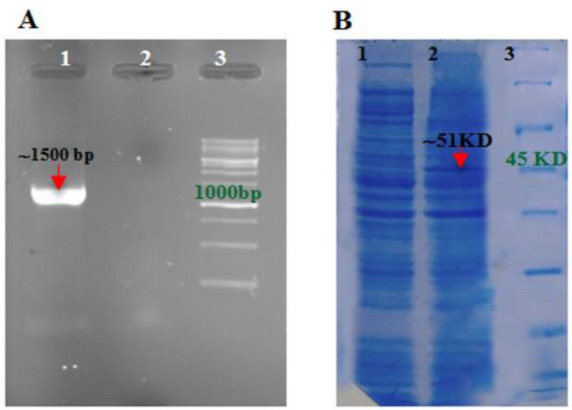
Gene Amplification and expression of Serra 470. A: Serra 470 gene amplification analyzed by electrophoresis on 1% agarose gel; (1) Serra 470, (2) Negative Control, and (3) DNA Ladder (Fermentas, 1 Kbp). B: SDS-PAGE analysis of Serra 470 expression using pET28a as vector; 1) before induction, 2) after induction with IPTG, and 3) protein marker (Fermentas, 14.4–116 kDa)

Amplification of Serra 470 gene using designed primers confirmed with detecting a DNA band at the expected size (∼ 1500 bp) on 1% agarose gel (Fig. 1A). Expression of recombinant Serra 470 in *E. coli* BL21 was approved by observing the protein band at the expected sizes (∼50 kDa) that revealed by SDS-PAGE (Fig. 1B). The amount of recombinant protein was very low. So further steps were performed to optimize the protein expression.

### Expression optimization of Serra 470 recombinant protein

To achieve high amount of recombinant protein, different parameters were changed and the results were analyzed to determined optimized conditions. The results of each optimization were summarized below:

### Expression vector replacement

**Fig. 2:**
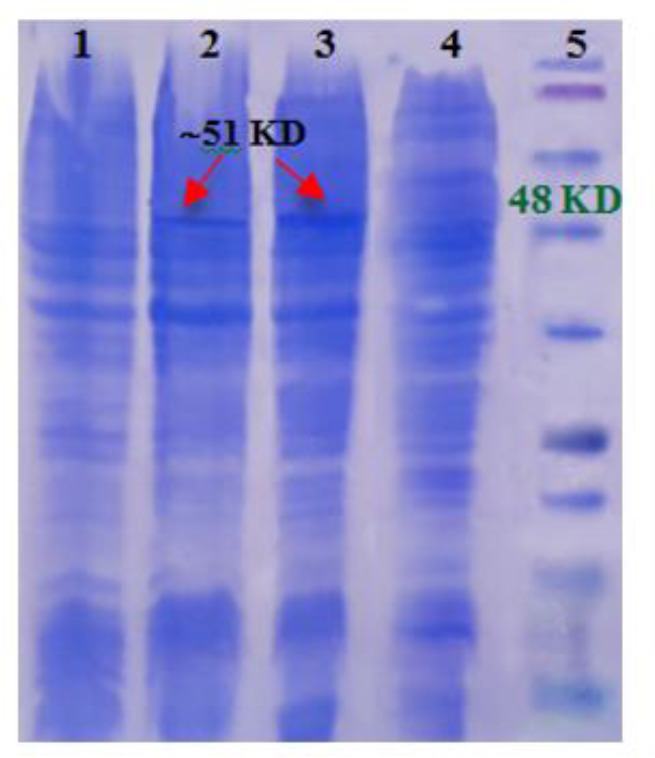
Comparing Serra 470 recombinant protein expression using two different vectors by SDS-PAGE; 1) Serra 470-pET28 before induction, 2) Serra 470-pET28 after induction, 3) Serra 470-pQE30 after induction 4) Serra 470-pQE30 before induction, and 5) protein marker (Fermentas, 11–245 kDa)

Transfection of Serra 470-pQE30 recombinant plasmids into *E. coli* M15 competent bacteria resulted in production of the recombinant protein with the expected size of ∼50 kDa as revealed by SDS-PAGE. Comparing the amount of expressed recombinant protein implementing two different vectors showed that Serra 470 was expressed more efficiently using pQE30 as vector (Fig. 2). The amount of expressed protein estimated by densitometry was 6.5% of total protein in contrast to using pET 28a as vector, which was about 4%. Therefore, in further experiments the *E. coli* M15 carrying Serra 470-pQE30 was used as the optimized host.

### Optimization of culture media

**Fig. 3:**
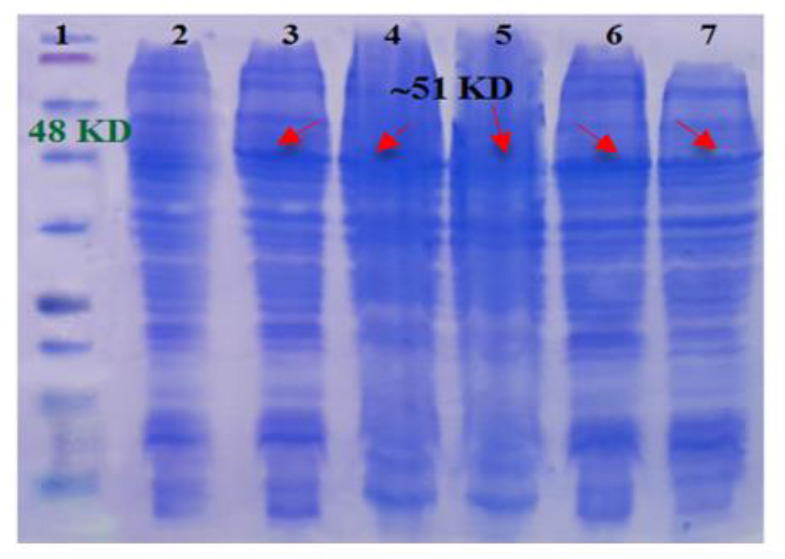
SDS-PAGE analysis of culture media optimization; 1) protein marker (Fermentas, 11–245 kDa), 2) 470-pQE30 before induction, 3) 470-pQE30 in SOB after induction, 4) 470-pQE30 in 1X LB after induction, 5) 470-pQE30 in 2X LB after induction, 6) 470-pQE30 in TB after induction, and 7) 470-pQE30 in 2XYT after induction

Different culture media were used for cultivation of *E. coli* M15 carrying Serra 470-pQE30 and the amount of protein expression was compared. The SDS-PAGE results illustrated that TB medium was the most appropriate than other examined media because the outcome of expressed recombinant proteins was higher using TB medium (Column 6, Fig. 3). Quantitatively the amount of recombinant protein calculated as 5.8%, 6.8%, 8.2%, 11%, and 12.8% of total proteins using 1X LB, 2X LB, SOB, 2X YT and TB media, respectively.

### Optimization of post-induction incubation temperature

**Fig. 4:**
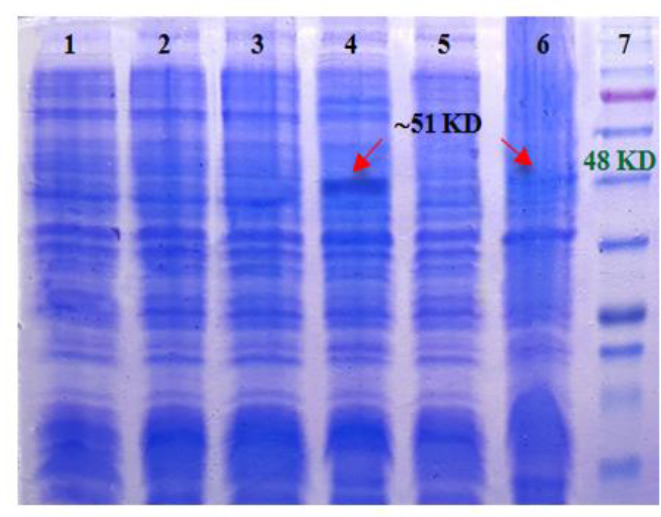
SDS-PAGE analysis of 470-pQE30 post-induction incubation temperature Optimization; 1,2) before and after induction with IPTG in 25 °C, respectively; 3,4) before and after induction with IPTG in 37 °C, respectively; 5,6) before and after induction with IPTG in 30 °C, respectively; 7) protein marker (Fermentas, 11–245 kDa)

After selecting the most appropriate culture media, Serra 470 was expressed in TB medium under different temperatures. Comparison of post-induction temperatures on the SDS-PAGE was shown that the highest expression level occurred at 37 °C (Fig. 4). No protein band was determined at 25°C, however there was a very weak protein band at 30 °C.

### Optimization of inducer concentration

**Fig. 5:**
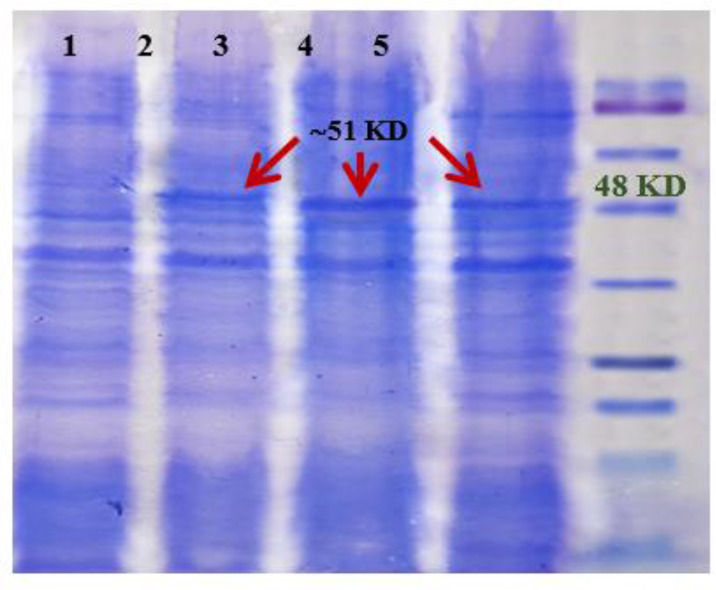
SDS-PAGE analysis of 470-pQE30 expression induced by different IPTG concentrations; 1) Bacterial pellet before induction; 2,3,4) Bacterial pellet after induction with 0.2 and 0.5 and 1 mM IPTG, respectively; 5) protein marker (Fermentas, 11–245 kDa)

Genes controlled by the lac or tac promoter/operator sequences are expressed to high levels in the presence of IPTG. However, since IPTG is an expensive chemical agent not metabolized by the host, it is strongly recommended to determine the efficient concentration of IPTG in each expression system. Therefore, in the present investigation different concentrations of IPTG have been studied and based on the SDS-PAGE results 0.5 mM (densitometry estimates the protein amount around 11.3%) was chosen as the most appropriate concentration for induction of serratiopeptidase (Fig. 5). However, the amount of recombinant protein was almost equal (11.5%), using 1 mM of IPTG but due to mentioned points, the 0.5 mM was selected as the most appropriate one.

### Optimization of post-induction incubation time

**Fig. 6:**
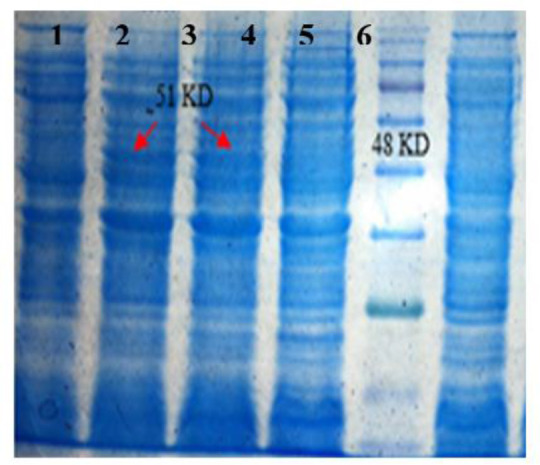
SDS-PAGE analysis of post-induction incubation time for optimization of Serra 470 expression; (1–4) post-induction incubation in 2 h, 4 h, 6 h and O.N., respectively, (5) protein marker (Fermentas, 11–245 kDa), and (6) 470-pQE30 before induction with IPTG

Comparison of different post-induction incubation times for Serra 470 expression in *E. coli* M15 (TB, 37 °C, 0.5mM IPTG) showed that the highest amount was obtained at 4 h (11.4%) and 6 h (11.5%) after induction with IPTG as analyzed by SDS-PAGE (Fig. 6). Considering limited time during practical studies, 4 h after induction was selected as the best post-induction incubation time.

### Confirmation of recombinant protein

**Fig. 7:**
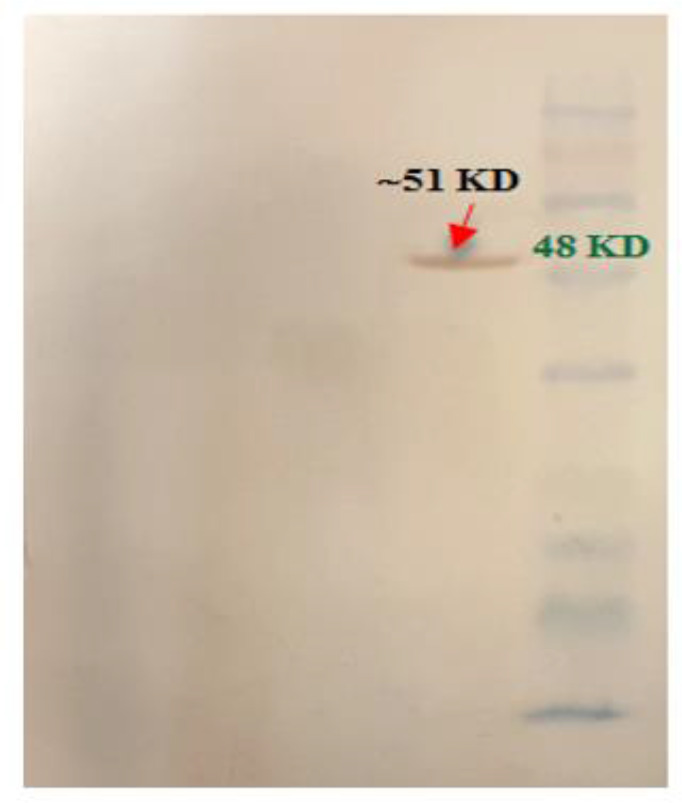
Western blot analysis of Serra 470. 1,2) Bacterial pellet carrying pQE30 without insert gene as a negative control, before and after induction with IPTG, respectively; 3,4) Bacterial pellet carrying Serra 470-pQE30 before and after induction with IPTG, respectively; and 5) protein marker (Fermentas, 11–245 kDa)

Finally, Serra 470 was expressed under optimized conditions (Table 1) and then verified by Western blot assay using anti-His antibody (Fig. 7).

**Table 1:** Summary of Serra 470 recombinant protein optimization

***Optimized parameters***	***Alternations***	***Amount of expressed protein (%)***	***Final optimized condition (%)***
Vector	pET28	46.5	pQE30 (6.5)
	pQE30		
Culture media	1X LB	5.8	TB (12.8)
	2X LB	6.8	
	TB	12.8	
	2X YT	11	
	SOB	8.2	
Post-induction incubation temperature			37°C (12.9)
	25 °C	1	
	30 °C	4.2	
	37 °C	12.9	
Inducer concentration	0.2 Mm	7.7	0.5 mM (11.3)
	0.5 mM	11.3	
	1 mM	11.5	
Post-induction incubation time	2 h	8.1	4 h (11.4)
	4 h	11.4	
	6 h	11.5	
	Overnight	3	

## Discussion

Serratiopeptidase that recently named as miracle enzyme has been used widely throughout Europe and Asia, as a feasible substitute to aspirin (salicylates), ibuprofen and the more potent NSAID drugs. It is a naturally occurring protease, which digests non-living tissue, blood clots, cysts, and arterial plaque and inflammation in all forms with no inhibitory effects on prostaglandins and is avoid of gastrointestinal side effects. This enzyme was initially found in the silkworm intestine but is manufactured commercially today through fermentation (15). Recombinant production of pharmaceutical proteins is another approach that is applicable to this enzyme as well.

For proteins that are not required post-translational modification, bacteria are an excellent expression system because of their relative simplicity, and the lower cost. In the present study, serratiopeptidase was expressed in *E. coli* in the form of inclusion bodies. Then different parameters were optimized to obtain the highest amount of expressed protein. pET 28a plasmid was selected as expression vector based on the previous study which also resulted in very weak protein band on the SDS-PAGE (16). Besides, in the present investigation by comparing expression level using two different vectors (pET 28a and pQE30), pQE30 was more effective in expression of the enzyme than pET 28a plasmid. In the next step, other expression parameters including different culture media, altered post-induction incubation temperature, various inducer concentration and post-induction incubation time were examined and optimized based on the previous studies conducted to elevate the amount of other recombinant proteins (17–19). Although expression was accomplished using variety of different media, amount of the recombinant protein was much higher in TB medium. For other parameters, the best condition was induction with 0.5 mM IPTG concentration, at 37 ºC and harvesting cells 4 h after induction. The optimal IPTG concentration and the temperature were in line with another study, which produced recombinant serratiopeptidase in *E.coli* for immunological studies (20). As the recombinant protein was confirmed by western blot assay, it could be considered for pharmaceutical applications in future studies.

## Conclusion

In the present investigation the thermostable form of serratiopeptidase, shown to have diverse clinical usages, expressed as recombinant protein in *E. coli*. Then different expression parameters were optimized to achieve the highest yield of the recombinant enzyme. The results of this study suggested that recombinant technology is also a putative way to produce this valuable therapeutic enzyme.

## Ethical considerations

Ethical issues (Including plagiarism, informed consent, misconduct, data fabrication and/or falsification, double publication and/or submission, redundancy, etc.) have been completely observed by the authors.
